# The potential monetary benefits of reclaiming hazardous waste sites in the Campania region: an economic evaluation

**DOI:** 10.1186/1476-069X-8-28

**Published:** 2009-06-24

**Authors:** Carla Guerriero, John Cairns

**Affiliations:** 1 London School of Hygiene and Tropical Medicine, London, UK

## Abstract

**Background:**

Evaluating the economic benefit of reducing negative health outcomes resulting from waste management is of pivotal importance for designing an effective waste policy that takes into account the health consequences for the populations exposed to environmental hazards. Despite the high level of Italian and international media interest in the problem of hazardous waste in Campania little has been done to reclaim the land and the waterways contaminated by hazardous waste.

**Objective:**

This study aims to reduce the uncertainty about health damage due to waste exposure by providing for the first time a monetary valuation of health benefits arising from the reclamation of hazardous waste dumps in Campania.

**Methods:**

First the criteria by which the landfills in the Campania region, in particular in the two provinces of Naples and Caserta, have been classified are described. Then, the annual cases of premature death and fatal cases of cancers attributable to waste exposure are quantified. Finally, the present value of the health benefits from the reclamation of polluted land is estimated for each of the health outcomes (premature mortality, fatal cancer and premature mortality adjusted for the cancer premium). Due to the uncertainty about the time frame of the benefits arising from reclamation, the latency of the effects of toxic waste on human health and the lack of context specific estimates of the Value of Preventing a Fatality (VPF), extensive sensitivity analyses are performed.

**Results:**

There are estimated to be 848 cases of premature mortality and 403 cases of fatal cancer per year as a consequence of exposure to toxic waste. The present value of the benefit of reducing the number of waste associated deaths after adjusting for a cancer premium is €11.6 billion. This value ranges from €5.4 to €20.0 billion assuming a time frame for benefits of 10 and 50 years respectively.

**Conclusion:**

This study suggests that there is a strong economic argument for both reclaiming the land contaminated with hazardous waste in the two provinces of Naples and Caserta and increasing the control of the territory in order to avoid the creation of new illegal dump sites.

## Background

Uncertainty regarding waste generation, waste management practices, data on emissions, exposure characterization and, in particular, the health risk associated with the different types of waste management methods is the main cause of the extensive market failure in the management of waste disposal. Several population studies document (scientifically) that the mismanagement of waste disposal can have serious effects on the health and well being of the population [1-4]. A wide range of toxic substances can be released into the environment from waste disposal, for example, methane, carbon dioxide, benzene and cadmium. Many of these pollutants have been shown to be toxic for human health. The International Agency for Research on Cancer [5] classifies exposure to cadmium and benzene as highly carcinogenic for humans. In addition, if the waste disposals are illegal, then they are likely to contain highly hazardous compounds resulting from industrial production, for example asbestos and lead [6].

Previous epidemiological studies have found that two main health outcomes – cancer and congenital malformations – are statistically associated with waste exposure [2-4,7,8]. Hazardous waste has been shown to influence the likelihood of developing brain, bladder and lung cancer [9,10]. According to Dolk *et al*. [11] living close to a waste disposal site is also associated with a significant increase in congenital anomalies. They report an odds ratio of 1.33 (CI: 95% 1.11–1.59, adjusted for socioeconomic status and maternal health) for congenital anomalies among those living within 3 km of hazardous waste (landfill) sites in Europe. Bentov *et al*. [12] also find a significantly increased risk of central nervous system malformations for those individuals living close to toxic waste sites (1.63 CI: 95% 1.34–1.80).

In the Campania region, in particular in the two provinces of Naples and Caserta, the absence of other types of waste management methods (composting, recycling, incinerators) and the extent of illegal toxic dumping of wastes are the main reasons for the waste crisis which was officially declared by the Consiglio dei Ministri in 1994 and since 2002 has become known worldwide as a "tragedy" [13,14]. Campania has the highest number of environmental crimes in Italy and it is estimated that 5 million tons of hazardous industrial residuals have been illegally discarded in the region [6,15-17]. According to WHO *et al*. [18] and Mutasem El-Fadel *et al. *[19] the waste-associated health hazards in this region have reached an unacceptable level and the problem now represents a real threat to human health.

Since the first research evaluating the relationship between waste exposure and an excess of early mortality and congenital malformation, an increasing number of studies report a statistically significant relationship between waste exposure and human health in Campania [20-24]. The most recent study conducted by WHO *et al*. [18] in 2007 documents higher rates of overall mortality, cases of fatal cancer and congenital malformations for those living in the area surrounding waste sites. For example, this study finds that women who live close to waste disposals classified as the most toxic have a 12% increased risk of dying and a 29% higher risk of developing liver cancer compared with those living in areas classified as environmentally safe.

To date, however, no studies evaluate the economic cost of ill-health associated with toxic waste exposure in this region. Thus, the aim of the present study is to estimate the potential benefit from reclaiming the landfill sites in Naples and Caserta provinces.

## Methods

**Figure 1:**
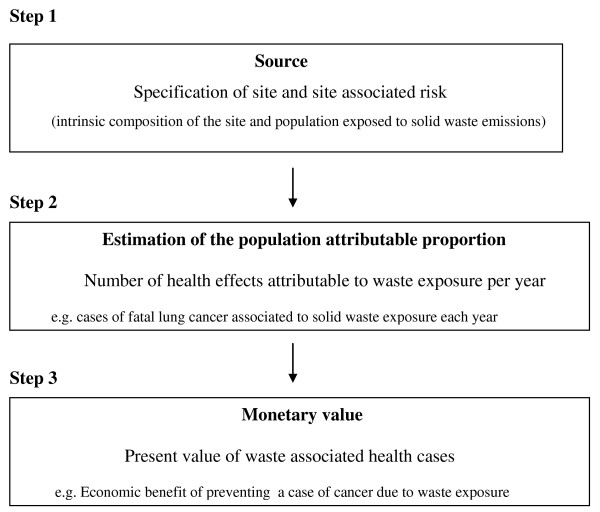
**Method used for evaluating the health**.

The present study spans three main fields: environmental externalities associated with waste management, epidemiology and economics. The three steps to assign a monetary value to the health benefits arising from the reclamation of hazardous waste sites in Campania are shown in the flow chart (Figure 1). The study starts with the criteria by which the Italian Protezione Civile classify the waste disposal sites in the provinces of Naples and Caserta using a Waste Index (WI) [18]. The second part of the study quantifies the annual physical impacts due to waste exposure. Health outcomes are estimated for each WI quintile using the exposure-response function from the WHO *et al. *[18] longitudinal study. Finally, the present value of the benefits over a period of 30 years arising from land reclamation is estimated. The monetary values used to assign a value to premature death and to a case of fatal cancer are selected according to European Commission (EC) recommendations and are adjusted for the risk context and for different time frames of the predicted health benefits in the sensitivity analysis [25].

### Classification of solid waste disposals in Naples and Caserta provinces

The impact chain shown in Figure 1 starts when waste enters the landfill or is abandoned illegally in the soil or in the water. Depending on the intrinsic quality of the waste and on the density of the population in the surrounding area hazardous emissions will be released into the environment affecting human health. According to previous studies in Campania, the health risk due to environmental hazards arising from waste exposure is confined to the two provinces of Naples and Caserta where most of the illegal dumping sites are located [6,13,15,18]. The dumping sites in Naples and Caserta differ in dimension and composition. In addition, most of these waste disposals are illegal and not visible (sunken or buried) thus the toxic substances that the disposal contains are not known and are difficult to identify. In order to map the possible areas exposed to a higher waste related health risk the Protezione Civile developed a synthetic index – the WI [13]. Using a GIS system the Protezione Civile identify the areas of waste impact in these two provinces and classify each of the 196 towns according to the number of waste disposals present, the intrinsic composition of the waste disposals and the proportion of the population living in the areas surrounding the dumping site [13]. The higher the presence of toxic waste sites/population exposed the higher is the WI assigned to the town [13].

### Estimation of the population attributable proportion due to waste exposure

In order to estimate the incremental health outcomes arising from waste exposure each year it is necessary to evaluate the gradient of the dose-response relationship between the WI and the health outcomes observed after controlling for the socioeconomic factors [18,26].

Several studies evaluating the effects of waste exposure on health in Campania find a statistically significant relationship between the presence of illegal dumping sites and higher prevalence of cancers and congenital malformations [18,20-23]. The most recent longitudinal study (WHO *et al*., [18]) analyses mortality records on twenty causes of death (e.g. all cause mortality, all types of cancers, lung cancer, liver cancer, stomach cancer, non Hodgkin lymphomas) for each of the 196 towns of the Caserta and Naples provinces between 1994–2001. The relative risks of different health outcomes given different levels of waste exposure are estimated by Poisson regression after controlling for socioeconomic factors [18].

The population attributable proportion (PAP) of the overall cases of premature mortality and fatal cases of cancer due to waste exposure are quantified using the results from this study. The number of cases (e.g. cancers) that would not have occurred in the absence of the environmental risk factor, for each health outcome and level of WI is estimated by the following formula:

Where *a *is the health outcome and *b *is the WI quintile considered and *Relative Risk*_*ab *_the relative risk of developing a given health outcome *a *(e.g. premature death) for each WI quintile *b *after controlling for socioeconomic factors [18,26,27]. Assuming that the effects of waste exposure on human health are equally distributed over time, the yearly number of health outcomes attributable to waste exposure is given by dividing the PAP of each health outcome by eight (the number of years of the longitudinal study).

### Monetary valuation of the health benefits arising from land reclamation

Assigning a monetary value to the health benefits arising from a reduction in the negative environmental externalities is not the same as placing a monetary value on a human life [27]. What is being evaluated in this study, in monetary terms, is the benefit of preventing the deaths attributable to waste exposure in the future. This study does not attempt to assign a monetary value to the several hundreds of deaths that have already occurred due to waste exposure in Campania since the creation of the first toxic landfills in the mid 1980s [15]. The Value of Preventing a Fatality, as the name suggests is how much individuals are willing to pay for reducing the risk of dying from a given environmental hazard. Thus, what is being evaluated in the present study is the benefit of reducing future deaths due to waste exposure.

According to the Enhealth-guidelines [28] there are two main methods for valuing health: the human capital and the willingness to pay approach. The human capital approach assumes that the value of an individual's life to the society can of be measured by future production potential, for example, future labour earnings. Based on the human capital approach, the Cost of Illness (COI) method measures *ex post *the costs arising from a specific negative health outcome, including the cost of hospitalization, medical consultations, and death [29]. Although this approach takes into account all the direct costs associated with a given disease it does not include the intangible costs: pain, discomfort and depression that are associated with an adverse health outcome and, especially for a very serious health outcome, it tends to underestimate the true cost of the disease [27]. Another weakness of the COI approach is that it is an *ex post *measure of costs and it does not consider the value that individuals give to possible risk reduction interventions [27].

For these reasons the WTP approach is adopted in this study. It is the most commonly used method in the evaluation of environmental health effects as it measures *ex ante *how much individuals are willing to pay for a reduction in the probability of an adverse event. Since the WTP approach has not been used to estimate the VPF in Italy, nor in the context of waste exposure, this study uses the VPF suggested by the EC [30]. These estimates: €3.7 million as an upper value, €1.4 million as a baseline estimate and €0.95 million as a lower value, are re-expressed in 2007 prices using the Harmonised Index of Consumer Prices [31]. There are two main benefits to using the values suggested by the EC [30]: they have been adjusted for the age of victims of environmental pollution and they can be applied to all the EU countries.

Several studies report that the VPF to avert a fatal case of cancer is higher than the VPF for reducing the risk of a death that is not proceeded by a long period of serious disability (e.g. fatal heart attack) [30,32,33]. Cancer is associated with a long period of serious illness and a high burden of pain and discomfort. Thus, because of the "dread" of such a long period of suffering, individuals tend to place a higher monetary value on averting a fatal case of cancer than a case of premature but less painful death. In order to account for the "cancer the  premium", that is increased WTP of individuals arising from the dread of the illness, EC [30] recommends that the value of preventing a statistical cancer-related fatality is 50% higher. Thus, the upper, baseline and lower estimates used in this study are € 5.55 million, € 2.1 million and €1.42 million respectively.

The formula used to estimate the present value of the health benefit arising from the reclaiming of polluted waste sites is reported below [34]. It treats *X*_a _the estimated annual number of health outcome *a *as an annuity lasting *t *years. This is re-expressed as a present value using the discount rate *d*. This future present value of an annuity is then itself discounted to take account of the latency period *l*, which is the time occurring between the reduction of the exposure and the improvement in the health of the population [35]. λ is the VPF for the health outcome *a*.

In the baseline scenario three assumptions are made: the benefit to human health from reclamation of waste sites lasts 30 years; the discount rate is 4 percent; and the latency period is 20 years. As per EC [25] recommendations sensitivity analyses are performed using different time frames for heath benefit arising from land reclamation (10, 20 and 50 years), a 2 per cent discount rate and different latency periods (10 and 30 years).

There are no epidemiological studies that evaluate the latency of toxic waste effects on human health. The presence of illegal toxic waste sites in Campania has been documented since the 1980s thus it is not possible to infer from the epidemiological study conducted by WHO *et al. *[18] whether the excesses in premature mortality and cancers are the consequence of a recent or long exposure to waste emissions. In the base case scenario it is assumed that the annual waste-associated deaths will disappear twenty years after land reclamation. In practice, it is likely that there will be a gradual decline in the number of waste associated health outcomes over time. As the speed of the reduction in deaths arising from waste reclaim is unknown, in the sensitivity analysis a 20% reduction in the number of deaths and fatal cancers is assumed from the sixteenth until the twentieth year.

## Results

### Number of waste attributable cases

The health outcomes attributable to waste exposure are shown separately for men and women. The estimated Relative Risk (RR) of developing the health outcome with respect to the first quintile (which contains the towns least exposed to negative externalities from waste exposure) is reported in the second column of Table 1. The third column contains observed cases of the health outcome from 1994–2001. The fourth column shows the estimated PAP. Only cases resulting from a statistically significant (p < 0.05) RR are considered. Finally the last row reports the number of cases attributable to waste exposure each year for both sexes.

**Table 1:** Number of fatal cases attributable to waste exposure.

	Waste index	Relative Risk	Observed cases 1994–2001	PAP^a ^1994–2001
**Male**				

	2	1.05^b^	53106	2528

	3	1.08^b^	7853	580

	4	1.04^b^	20130	774

	5	1.08^b^	8459	698

**Female**				

	2	1.02	52167	1023

	3	1.08^b^	7124	528

	4	1.05^b^	18226	868

	5	1.12^b^	7501	804

**Cases over 8 year follow-up**				**6780**
**Cases over 1 year**				**848**

**a **Population attributable proportion over 8 year follow up [18].

**b **p value < 0.05

According to the WHO *et al. *[18] study, men living in the second, third, fourth and fifth WI quintiles have 5%, 8%, 4% and 8% higher risk of dying compared to men living in the areas least exposed to waste. Women are even more exposed to the effects of waste than men as the RRs for each WI class are higher compared to men in all quintiles except the second. Of the 89,530 deaths observed among men in these four quintiles between 1994–2001, 4,580 are associated with waste exposure. Among women the overall number of deaths is 85,018 and the number of waste attributable deaths is 2,200. The total number of fatal cases attributable to waste exposure each year in the two provinces of Naples and Caserta is 848.

Among men an increased risk of developing a case of fatal cancer is observed across all the four quintiles with the exception of the fifth where the risk is not statistically significant (Table 2). Among women only those living in the towns included in the second and the fifth quintiles show an increased risk of dying by 5% and 7% respectively. The overall number of cancers observed is 72,674 of which 3,222 are attributable to waste exposure over an eight year period resulting in an estimate of 403 cases per year.

**Table 2:** Number of fatal cases of cancer attributable to waste exposure.

	Waste index	Relative Risk	Observed cases 1994–2001	PAP^a ^1994–2001
**Male**				

	2	1.04^b^	15989	615

	3	1.06^b^	2297	1658

	4	1.05^b^	6261	298

	5	1.04	2525	97

**Female**				

	2	1.05^b^	11435	544

	3	1.02	1490	30

	4	1.04	4038	155

	5	1.07^b^	1639	107

**Cases over 8 year follow-up**				**3222**
**Cases over 1 year**				**403**

**a **Population attributable proportion over 8 year follow up [18].

**b **p value < 0.05

### Health benefits arising from hazardous waste reclamation

Table 3 reports the present value of the health benefits attributable to reclamation of waste sites assuming benefits arise over a 30 year time frame, a 4% discount rate and 20 years of latency. All costs are reported in Euros in 2007 prices. The yearly population attributable proportion (for both sexes) is reported for each of the health outcomes.

**Table 3:** Monetary benefits arising from waste sites reclaim.

Item	PAP per year	Benefits^a ^(billion €)	Benefits per person(€) ^b^
All causes mortality	848	9.4 (6.3–25.0)^c^	2,300 (1,600–6,200)

All fatal cancers	403	6.7	1,700
		(4.5–17.0)^c^	(1,100–4,400)

All cause mortality adjusted for cancer premium	848	11.6 (30.4–7.8)^c^	3,000 (2,000–7,700)

a benefits have been rounded to the nearest million.

b benefits per person have been rounded to the nearest hundred

c Lower and Upper and estimates obtained using lower and upper values suggested by the EC [25].

The overall number of waste related deaths (from all causes) per year is 848. The overall benefit given the base case assumptions is €9.4 billion. Although the cases of fatal cancer are significantly lower (less than 50% of the all cause deaths) the overall benefit of preventing 403 fatal cancer cases associated with waste exposure is high: (€6.7 billion). Since the estimated €9.4 billion benefit of reducing 848 deaths does not account for the higher value assigned by individuals to deaths from cancer, a third estimate adjusted for the "cancer premium" is calculated. Further, the benefit per capita (rounded to the nearest thousand) of land reclamation is estimated by dividing the monetary benefit by the population living in the two provinces of Naples and Caserta in 2008 [36].

### Sensitivity analysis

**Figure 2:**
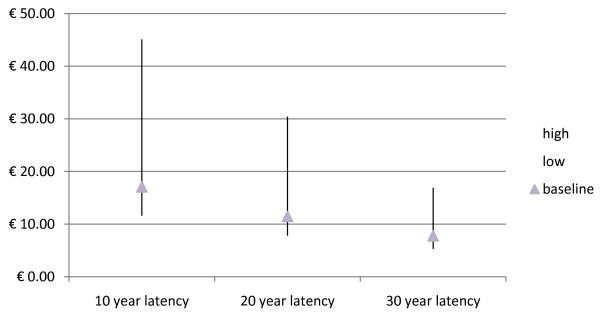
**Monetary benefits (billion€) assuming different latency periods and a 4% discount rate**.

**Figure 3:**
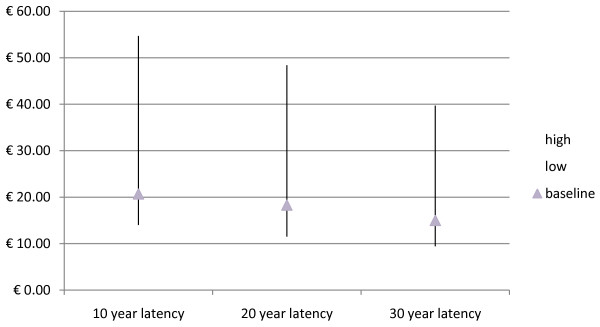
**Monetary benefits (billion€) assuming different latency periods and a 2% discount rate**.

Different assumptions about the latency of the effect of the pollutants and about the discount rate lead to different conclusions about the overall effects of toxic waste on human health. Health benefits arising from land reclamation in Campania are reported below assuming different time frames over which benefits are produced (Table 4). In Figures 2 and 3 the present value of the benefit of reducing the number of waste associated deaths after adjusting for cancer premium is reported assuming different latency periods and discount rates. Table 5 reports the monetary benefits assuming that during the latency period, from year sixteen until year twenty, the number of waste deaths and fatal cancers will decline annually by 20% as a result of the reclamation of waste sites.

**Table 4:** Monetary benefits (billion €) by time horizon over which benefits accrue^a^.

Item	50 year time	20 year time	10 year time
All causes mortality	17.0 (8.0–31.0)^b^	7.0 (5.0–19.0)^b^	4.0 (3.0–12.0)^b^

All fatal cancers	8.0 (6.0–22.0)^b^	5.0 (3.0–14.0)^b^	3.0 (2.0–8.0)^b^

All cause mortality adjusted for cancer premium	20.0 (9.7–38.0)^b^	9.0 (6.1–24.0)^b^	5.4 (3.7–14.3)^b^

a all costs have been rounded to the nearest million

b Upper and Lower estimates obtained using upper and lower values suggested by the EC [25]

**Table 5:** Monetary benefits (billion €) after accounting for the decline deaths during the after latency period.

Item	Benefits ^a^
All causes mortality	10.6 (7.1–28.0)^b^

All fatal cancers	7.5 (5.1–19.7)^b^

All cause mortality adjusted for "cancer premium"	12.8 (8.7–33.7)^b^

a all benefits have been rounded to the nearest million

b Upper and Lower estimates obtained using upper and lower values suggested by the EC [25]

## Discussion

Public awareness of the long term health effects associated with negative environmental externalities is increasing as a consequence of better data collection and the increasing number of epidemiological studies [37]. Assigning a monetary value to health risks arising from environmental externalities allows all the environmental influences on human health to be formally quantified and should help decision makers deliver optimal policies aimed at reducing the "external cost" to society. There is growing interest in the use of cost benefit analysis for the evaluation environmental interventions aimed at reducing the health damage associated with health pollution. Despite this, little has been done to quantify the external costs due to environmental hazards on health although they account for a large part of the damage associated with negative externalities.

Most of the studies, conducted in both developed and developing countries, on the effects of environment on human health focus on the long term effects of air pollution on mortality and morbidity and little attention is paid to evaluating the economic costs of waste-related health effects [27,38,39]. The results of the WHO *et al*. [18] study conducted in the Campania region suggest that in Naples and Caserta, the presence of toxic waste disposals is associated with an increased level of mortality, fatal cancers and some types of congenital malformations. Using WHO *et al*. [18] data this study estimates that between 1994 and 2001 6,781 of the overall 174,500 deaths in the region were associated with waste exposure. Looking at the specific causes of death, 3,222 fatal cancers in the eight year follow up of the WHO *et al*. [18] study are estimated to be associated with waste exposure – an estimated 403 cases per year. Using the VPF estimates suggested by the EC for environmental cost-benefit analyses, the present value of the health benefits arising from the reclamation of waste sites in the provinces of Naples and Caserta is €11.6. billion

This study makes several assumptions. According to the Protezione Civile [13] the potentially toxic waste sites located in the Campania region are concentrated in the area of Naples and Caserta provinces. However, it is very likely that there are other sites outside this area that are not documented so the problem is likely to have been underestimated. In terms of epidemiology, the specific effects of the single pollutants on health are not considered thus the transferability of the results of the present study to contexts other than the Campania region is limited.

Another important assumption of this study is that the relative risks used to quantify the number of deaths and fatal cancers attributable to waste exposure are estimated accounting for all the potential confounders. The WHO *et al. *[18] study controls for the socioeconomic gradient of the population living close to hazardous waste sites, however, important elements such as smoking rates are not accounted for and this could lead to the number of deaths and cancers being overestimated in the present study.

In addition, the health related effects considered are only the long term effects arising from waste exposure (death and cancer). Although, several short term effects are associated with toxic waste exposure such as malformations, asthma and respiratory infections these are not considered in the economic evaluation [11,12,27,40]. As a consequence, the potential benefit arising from land reclamation could be underestimated.

The EC [25] recommended values (upper, baseline and lower value) used are adjusted for the age of mortality of victims of environmental pollution and they provide a better estimate compared to previous VPF studies, however, were not elicited in the context of waste associated health risk. Further research is needed to provide a more comprehensive evaluation of the health effect arising from waste exposure and estimates of the VPF due to waste exposure.

As EC [25] suggested the majority of the research conducted in the field of waste focuses only on the tangible cost of methods of waste management and not the intangible benefits that can result. As long as the real costs and benefits of waste management policies, including their impact on health, are not explicitly accounted for in economic evaluations, there is a risk that poorer policies will be adopted and better policies rejected.

This study suggests that there is a high economic incentive to reclaim the hazardous waste in the two provinces of Naples and Caserta. According to "Protocollo Di Intesa" made by both the Italian Department for Environmental Safety (Ministero dell' Ambiente della Tutela del Territorio e del Mare) and the Campania Region, an investment of €143 million is required to reclaim the area of "Litorale Domizio and Agro Vesuviano", where the majority of the hazardous waste sites are located. This sum is dramatically lower than the estimated present value of the benefit of reducing the number of waste associated deaths – 11.6 billion [41].

## Conclusion

In recent decades the newly created illegal sites, existing illegal sites used as provisional landfills for the municipal waste, together with the increasingly popular practice among local criminal organizations of burning the toxic waste has produced annual increases in the number of waste-related health outcomes [6,15,42]. Consequently the potential monetary benefit from greater territorial control of waste sites and from employing reliable firms to perform reclamation of hazardous sites has increased. Neglecting the potential monetary benefits of reclaiming hazardous waste in Campania will result in further (tangible and intangible) costs for the Italian health care system and for those individuals that experience premature mortality and/or a long period of severe morbidity.

## Abbreviations

COI: cost of illness; EC: European Commission; EU: European Union; PAP: population attributable proportion; RR: relative risk; VPF: value of preventing a fatality; WPT willingness to pay; WI: waste index

## Competing interests

The authors declare that they have no competing interests.

## Authors' contributions

CG performed the literature review, drafted the manuscript and carried out part of the analysis. JC contributed substantially to defining the methods of the analysis, interpreting the results of the study and revising the manuscript for publication. Both authors read and approved the final version.
